# The Rinderpest

**Published:** 1866-04

**Authors:** John H. Griscom

**Affiliations:** London


### THE RINDERPEST.

LONDON, Jan. 11, 1866.

*Editor Philadelphia Medical and Surgical Reporter:*—An animated discussion of a very important subject is now going on among the professional and lay public. I allude to the nature and treatment of the *rinderpest*, or cattle disease, which has within a few months past created very extensive havoc, and more extensive alarm, over a very large portion of this kingdom, but especially in England. To such an extent has it prevailed in the vicinity of London, that the supply of milk is said to have been materially diminished. According to official reports made to the Government, the disease has attacked, during the last seven months of 1865, 73,549 out of a total number of 159,710 head of cattle in farms, sheds, and other places, where it was reported to exist. Of the whole number attacked, only 9579 recovered, about one-eighth.

Some eminent medical men have gone into an investigation of the character of the disorder. Mr. Ceely has recently discovered some points of analogy between human small-pox and rinderpest, and Dr. Murchison, the distinguished writer on fevers, has given an opinion confirmatory of this diagnosis. A medical report of 1839 is quoted from the Transactions of the Provincial Medical and Surgical Association, to prove "it has been shown by unquestionable evidence that cattle and other animals have for centuries been known to be affected with variola." It raged in England in 1745, and again in 1770, and it appeared amongst horned cattle, with more or less virulence, so late as 1780. Dr. Layard's observations on the successful inoculation from cow to cow is regarded as completing the chain of evidence in favor of the view that "it was the remains of this violent epizootic that Dr. Jenner found in Gloucestershire, and which, being occasionally transferred to the milkers, secured them from subsequent small-pox." What a treat the medical world is promising us! We have to pay dearly for it; but natural cow-pox has been a rarity for a long time past, and it is said to be possible—some declare probable—that the deadly murrain now afflicting our kine may turn to that human-life-preserving, benignant, and most desirable affection—cow-pox.

Another important witness in favor of the identity of variola and rinderpest, is Prof. Ferguson, in his report to the Government on the cattle plague in Ireland, as follows:—

He says:—"The results of some investigations recently made relative to the sanitary state of cattle that had had the pustular disease generally known as 'cow-pox' induce me to recommend that all bovine animals be inoculated with vaccine matter. As yet, there has not been sufficient direct evidence furnished by specially instituted experiments to justify the assertion that vaccination will insure cattle an immunity from the plague, or even a diminution of its virulence; but as vaccination has very rarely proved injurious to stock, and certainly protects it from some diseases, it is desirable that it be immediately had recourse to in Ireland, as an expedient that may possibly eventually prove of service should cattle-plague unfortunately make its appearance in this country. The milk of cows suffering from cow-pox should not be used as food either for human beings or calves."

The Pathological Society had the subject before them last week, and from the remarks made, it appears that a difference of opinion yet prevails upon it.

"Dr. Quain introduced Mr. Hancock, a veterinary inspector of the Uxbridge district, as the first individual brought under the notice of medical men with an eruption, 'the appearances of which were exactly those of a declining vaccine vesicle.' Dr. Murchison, Mr. Ceely, and Prof. Spooner were struck with the 'vaccine character' of the malady; and the first to discover the nature of this interesting case was Mr. Rayner, a surgeon at Uxbridge. Dr. Burdon Sanderson and Professor Gamgee spoke strongly against the probabilities of rinderpest being *variola*, and the first named gentleman has communicated an interesting description of the cattle-plague eruptions to the *Lancet*. He says:—'During the last month I have had opportunities of carefully observing the appearances presented by the cutaneous eruption in all its forms, and have watched it with all the attention of which I am capable. Without unduly anticipating the description of the skin-affection contained in my preliminary report, which is now ready to be submitted to the Royal Commission, I may state that the so-called scabs are not produced by the desiccation of pustules, but by the incrustation of the abundant secretion from the sebaceous follicles; that vesicles never occur at any period of the disease, the so-called flattened vesicles, described by Dr. Murchison, on the udder, being solid elevations, the structural elements of which are epidermal. The affection may be stated, so far as the investigations hitherto made enable me to judge, to be of the following nature:—There is, in the first place, hyperæmia of the cutis in the affected parts. Secondly, an excessive secretion from the cutaneous glands. Thirdly, an exuberant development of nuclear corpuscles in the deep layer of the epidermis, by which the more superficial layers are separated from the true skin. In some parts, particularly on the udder, near the roots of the teats, and on the scrotum, this exuberant growth of nuclear bodies takes place at particular points only, giving rise to the elevations above mentioned. These elevations, which have been accurately designated by previous authors as nodules (*knötchen*), or heaps (*höckerchen*), and other words implying their solid character, gradually soften and break up, sometimes becoming semi-fluid, and thus acquiring an unreal resemblance to pustules."

"Dr. Parkes, of Netley, writing in vindication of Mr. Ceely's claims to priority in the supposed discovery now agitating the public mind, says:—"The identity of cattle-plague and small-pox, though probable, is not yet proved. We must all anxiously wait for the experimental evidence which can alone definitely settle this momentous question.'"

From this it would appear that the question is yet an open one, but as the government has taken the matter in hand, energetically, it is to be hoped that it will receive the attention of the best diagnosticians of both the medical and veterinary schools. Respecting the treatment of the disease, while its real nature is undetermined, that must of course remain purely empirical and uncertain. Homœpathy has ventured upon this field also, but judging from the statements contained in the newspapers, its success has been coincident with the dimensions of its doses.

*Apropos* here, I enclose an article from the *London Punch* of this week, which is clearly to the point:—

TRICHIASIS.—That other serious disease of the brute creation, *Trichiasis*, which not only affects the hog, but also the human being, is likewise exciting a great deal of attention, more especially on the continent. Its ravages appear to have been recently quite extensive, and to have proved fatal in a number of instances among eaters of pork. It has been suggested as a necessary sanitary measure, that no pork, in any form, be allowed to be sold without having been first examined by the microscope, as the only means by which the presence of the *trichina spiralis* can be detected. Such a measure would seem to be imperative in places where the disease prevails—and as a prudential step it would be justifiable in many other localities.

Very truly yours,

JOHN H. GRISCOM.

